# Mindfulness for reducing stress, anxiety, and depression in incarcerated individuals: a scoping review

**DOI:** 10.15649/cuidarte.5159

**Published:** 2026-10-22

**Authors:** Yadira Mateo-Crisóstomo, Doriam Esperanza Camacho Rodríguez, Herlinda Aguilar Zavala, José Manuel Herrera Paredes, Jesús Antonio Zamorano Espero

**Affiliations:** 1 Universidad de Guanajuato, Celaya, Mexico. Universidad Juárez Autónoma de Tabasco. Villahermosa, Mexico. E-mail: y.mateocrisostomo@ugto.mx Universidad de Guanajuato Villahermosa Mexico y.mateocrisostomo@ugto.mx; 2 Universidad Cooperativa de Colombia. Santa Marta, Colombia. E-mail: doriam.camacho@ucc.edu.co Universidad Cooperativa de Colombia Santa Marta Colombia doriam.camacho@ucc.edu.co; 3 Universidad de Guanajuato, Celaya, Mexico. E-mail: h.aguilar@ugto.mx Universidad de Guanajuato Celaya Mexico h.aguilar@ugto.mx; 4 Universidad de Guanajuato, Celaya, Mexico. E-mail: mherrera@ugto.mx Universidad de Guanajuato Celaya Mexico mherrera@ugto.mx; 5 Universidad de Guanajuato, Celaya, Mexico. E-mail: ja.zamoranoespero@ugto.mx Universidad de Guanajuato Celaya Mexico ja.zamoranoespero@ugto.mx

**Keywords:** Mindfulness, Stress, Anxiety, Depression, Prisoners, Atención Plena, Estrés, Ansiedad, Depresión, Prisioneros, Atenção Plena, Estresse, Ansiedade, Depressão, Prisioneiros

## Abstract

**Introduction:**

Mental disorders affect one in eight people worldwide and cause significant disturbances in thinking, emotional regulation, and behavior.

**Objective:**

To map the effect of mindfulness intervention in reducing stress, anxiety, and depression levels in people deprived of their liberty.

**Materials and Methods:**

The five-stage methodological framework proposed by Arksey and O’Malley was used. A systematic literature search was conducted in the PubMed, SAGE Journals, ScienceDirect, Springer, and Web of Science databases.

**Results:**

A total of 10 articles were included, which analyzed 10 different interventions. Regarding participant sex, most studies focused on male populations, except for three studies that evaluated women and one that included a mixed-sex sample. Six of the seven interventions reviewed showed a significant reduction in at least one of these variables, with the Path of Freedom intervention standing out.

**Discussion:**

The adapted Path of Freedom program stood out as the most effective intervention for reducing stress, anxiety, and depression. This result suggests that its impact goes beyond traditional mindfulness practices by integrating key components of emotional regulation in a more structured way.

**Conclusion:**

Mindfulness-based interventions have proven to be effective in reducing stress, anxiety, and depression among incarcerated individuals. However, their impact varies depending on the methodological approach, program duration, and the components incorporated.

## Resumen

**Introducción:**

Los trastornos mentales afectan a una de cada ocho personas en el mundo y provocan alteraciones significativas en el pensamiento, la regulación emocional y la conducta.

**Objetivo:**

Mapear el efecto de la intervención de mindfulness en la reducción de los niveles de estrés, ansiedad y depresión en personas privadas de la libertad.

**Materiales y métodos:**

Se utilizó el marco metodológico de cinco etapas de Arksey y O´Malley. Se realizó una búsqueda sistematizada en las bases de datos PubMed, SAGE Journals, Science, Springer y Web of Science.

**Resultados:**

En total, se incluyeron 10 artículos, los cuales analizaron 10 intervenciones distintas. En cuanto a la distribución por género, la mayoría de los estudios se enfocan en poblaciones masculinas, con excepción de tres estudios que evaluaron a mujeres y uno que incluyó una muestra mixta. Seis de las siete intervenciones revisadas mostraron una disminución significativa en al menos una de estas variables, destacando la intervención Path of Freedom.

**Discusión:**

La adaptación del programa Path of Freedom destacó como la más efectiva en la reducción del estrés, la ansiedad y la depresión. Este resultado sugiere que su impacto va más allá de las prácticas tradicionales de Mindfulness, integrando de manera más estructurada componentes clave de la regulación emocional.

**Conclusiones:**

Las intervenciones basadas en Mindfulness han demostrado ser efectivas para reducir el estrés, la ansiedad y la depresión en personas privadas de la libertad. Sin embargo, su impacto varía según el enfoque metodológico, la duración del programa y los componentes incorporados.

## Resumo

**Introdução:**

Transtornos mentais afetam uma em cada oito pessoas no mundo e causam alterações significativas no pensamento, na regulação emocional e no comportamento.

**Objetivo:**

Mapear o efeito da intervenção de mindfulness na redução dos níveis de estresse, ansiedade e depressão em indivíduos encarcerados.

**Materiais e métodos:**

Foi utilizado o modelo metodológico de cinco etapas de Arksey e O'Malley. Uma busca sistemática foi realizada nas bases de dados PubMed, SAGE Journals, Science, Springer e Web of Science.

**Resultados:**

Um total de 10 artigos foram incluídos, analisando 10 intervenções diferentes. Em relação à distribuição por gênero, a maioria dos estudos focou em populações masculinas, com exceção de três estudos que avaliaram mulheres e um que incluiu uma amostra mista. Seis das sete intervenções revisadas mostraram uma diminuição significativa em pelo menos uma dessas variáveis, com a intervenção Caminho da Liberdade se destacando.

**Discussão:**

O programa Caminho da Liberdade adaptado se destacou como o mais eficaz na redução do estresse, da ansiedade e da depressão. Este resultado sugere que seu impacto vai além das práticas tradicionais de mindfulness, integrando componentes-chave da regulação emocional de forma mais estruturada.

**Conclusões:**

Intervenções baseadas em mindfulness têm se mostrado eficazes na redução do estresse, da ansiedade e da depressão em indivíduos encarcerados. No entanto, seu impacto varia dependendo da abordagem metodológica, da duração do programa e dos componentes incorporados.

## Introduction

Mental health has become the leading global health concern since 2021^1^,^2^. Conditions such as depression and anxiety disorders affect 3.8% and 4.0% of the global population, respectively^3^,^4^. In Latin America and the Caribbean, anxiety disorders have the highest prevalence worldwide, while depressive disorders rank second^5^.

Mental health problems have a greater impact on vulnerable populations, such as incarcerated individuals^6^, because they are exposed to a highly stressful environment^7^ characterized by loss of autonomy, judicial uncertainty, the need to adapt to strict institutional rules^8^, overcrowding^9^, violence, lack of privacy, and interpersonal conflicts^10^, as well as disconnection from the family environment and the absence of a sense of purpose^11^.

Stress, anxiety, and depression are interconnected and may reinforce one another over time^12^. Although negative emotions serve an adaptive function by helping individuals identify threats, their persistence may trigger maladaptive responses and increase the risk of mood disorders^13^. Perceived stress arises from the appraisal of a situation as threatening and the perception that one lacks sufficient resources to cope with it^14^. Its persistent presence may lead to anxiety, characterized by a state of heightened alertness and recurrent negative thoughts, which, in turn, further amplifies the perception of stress through a feedback loop^15^.

Stress is a key factor in the development of depression, particularly among vulnerable populations exposed to overcrowding, violence, and uncertainty^16^. The diathesis-stress model posits that certain stressful situations may trigger depressive episodes, while persistent depression may deplete cognitive resources, further impairing the ability to cope with stress^17^. This process creates a downward spiral in which stress accumulates and exacerbates emotional disorders^18^.

One strategy for interrupting this cycle is mindfulness^19^, for which evidence supports its effectiveness in reducing stress, anxiety, and depression by promoting adaptive coping strategies^20^. In correctional settings, its implementation has demonstrated benefits such as enhanced resilience, improved impulse control, and reduced aggressive behavior^21^,^22^.

Evidence regarding the effectiveness of mindfulness in correctional settings remains limited and fragmented^23^, although studies have examined its effects on emotional regulation and the reduction of aggressive behavior^24^. The objective of this scoping review was to map the effects of mindfulness interventions on reducing levels of stress, anxiety, and depression among incarcerated individuals.

## Materials and Methods

This scoping review^25^ was conducted using the five-stage methodological framework developed by Arksey and O’Malley^26^. The study findings are reported in accordance with the PRISMA-ScR 2020 (Preferred Reporting Items for Systematic Reviews and Meta-Analyses Extension for Scoping Reviews) reporting guidelines^25^.

**Research question**

The research question that guided the search strategy was developed using the PICO framework (P = Patient or Problem, I = Intervention, C = Comparison, and O = Outcome)^27^. The population of interest consisted of incarcerated individuals, the primary intervention was mindfulness, and the outcome variables included stress, anxiety, and depression. The following key questions guided the review:

What is the scope of the evidence on the effects of mindfulness interventions in reducing stress, anxiety, and depression among incarcerated individuals?

What characteristics of mindfulness interventions have been shown to be effective in reducing stress, anxiety, and depression in this population?

**Relevant studies**

A systematized literature search was conducted in the PubMed, SAGE Journals, ScienceDirect, Springer, and Web of Science databases between January and March 2025. The search strategy included MeSH and DeCS terms in Spanish, English, and Portuguese:“Mindfulness,”“Prisoners,”“Stress, Psychological,” “Anxiety,” “Depression,” “atención plena,” “prisioneros,” “estrés psicológico,” “ansiedad,” “depresión,” “Atenção Plena,” “Prisioneiros,” “Estresse Psicológico,” “Ansiedade,” and “Depressão.” These terms were combined using the Boolean operator AND. The search strategy was adapted to the specific requirements of each database and tested in all three languages before the final search was conducted.

**Study selection**

Original articles reporting randomized controlled trials, as well as studies reporting a single randomized or quasi-experimental clinical trial, published between January 1, 2015, and January 1, 2025, were included. Eligible studies had to evaluate mindfulness interventions in relation to stress, anxiety, and depression, be available through open access, and be published in Spanish, English, or Portuguese. Studies that did not report outcome measures for any of these dimensions were excluded, as were non-systematic literature reviews, study protocols, gray literature (e.g., conference abstracts and theses), and descriptive or correlational studies.

**Data charting**

The study selection process was conducted sequentially, from identification through inclusion, following the PRISMA-ScR flow diagram^25^. Initially, studies were identified through searches of the PubMed, SAGE Journals, ScienceDirect, Springer, and Web of Science databases, as well as through manual searches. Duplicate records were then removed using reference management software. Subsequently, the records were organized in a Microsoft Excel spreadsheet to assess their eligibility.

Two reviewers independently screened the titles and abstracts by applying the eligibility criteria. Studies considered potentially relevant were retrieved in full text for further assessment. Reasons for exclusion were documented for subsequent review by a third reviewer. Finally, any disagreements arising during the final stage of the selection process were resolved by the third reviewer, leaving no unresolved discrepancies.

**Collating, summarizing, and reporting the results**

Using a standardized data extraction form, two reviewers independently extracted the data and organized them in a Microsoft Excel spreadsheet. The extracted information included the year of publication, the country where the study was conducted, the sample size, and specific data on the characteristics of the mindfulness intervention. In addition, the interventions were critically appraised using the TIDieR (Template for Intervention Description and Replication) checklist to evaluate the completeness of intervention reporting and the replicability of each intervention.

The 12 checklist items were independently assessed by two reviewers, who evaluated aspects such as the intervention name, theoretical foundation, materials, procedures, facilitator qualifications, mode of delivery, setting, duration, adaptations, and intervention fidelity. The results were organized into an Excel matrix to facilitate comparative analysis. All extracted data are publicly available through Mendeley Data^28^.

**Ethical considerations**

Review studies do not require approval from a research ethics committee because they do not involve the direct collection of data from participants. However, they must adhere to a rigorous methodological approach to ensure the validity and reliability of the findings.

## Results

A total of 628 records reporting interventions described in original articles were identified. After duplicate records were removed, an initial screening based on titles and abstracts was conducted, resulting in the exclusion of 545 records that did not meet the eligibility criteria. As a result, 80 articles were selected for full-text review.

In addition, a complementary search was conducted to identify randomized controlled trials or quasi-experimental studies that specifically evaluated mindfulness interventions. Of the studies retrieved, 60 were excluded because they did not meet the predefined eligibility criteria. The main reasons for exclusion included the absence of a focus on mindfulness-based interventions, theoretical papers or review articles without empirical findings, inclusion of populations other than the target population, insufficient methodological information, and unavailability of the full text. Ultimately, 20 articles were identified as potentially relevant, of which 10 met all the established eligibility criteria and addressed a range of mindfulness interventions Figure 1.

To address the research questions, the selected studies were analyzed systematically, and their findings were classified into three main domains: (1) characteristics of the participant population, (2) study design and intervention components, and (3) study results and the effects of the intervention on stress, anxiety, and depression.

**Characteristics of the study population**

Table 1 summarizes the characteristics of the study populations included in the selected studies published between 2015 and 2025. These studies were conducted across diverse regions of the world, including the United States^29^,^30^, China^31^-^33^, the Netherlands^34^, the United Kingdom^35^,^36^, Spain^37^, and Portugal^38^, providing a global perspective on the use of mindfulness interventions in correctional settings.

Regarding participant sex, most studies focused on male populations, with the exception of three studies that evaluated women and one that included a mixed-sex sample. Participants’ ages varied widely, ranging from adolescents aged 14 years to adults aged 60 years, with study-specific differences according to the geographic setting and study design. Marital status was reported in only a few studies, indicating that participants were either single or married.

**Figure 1:**
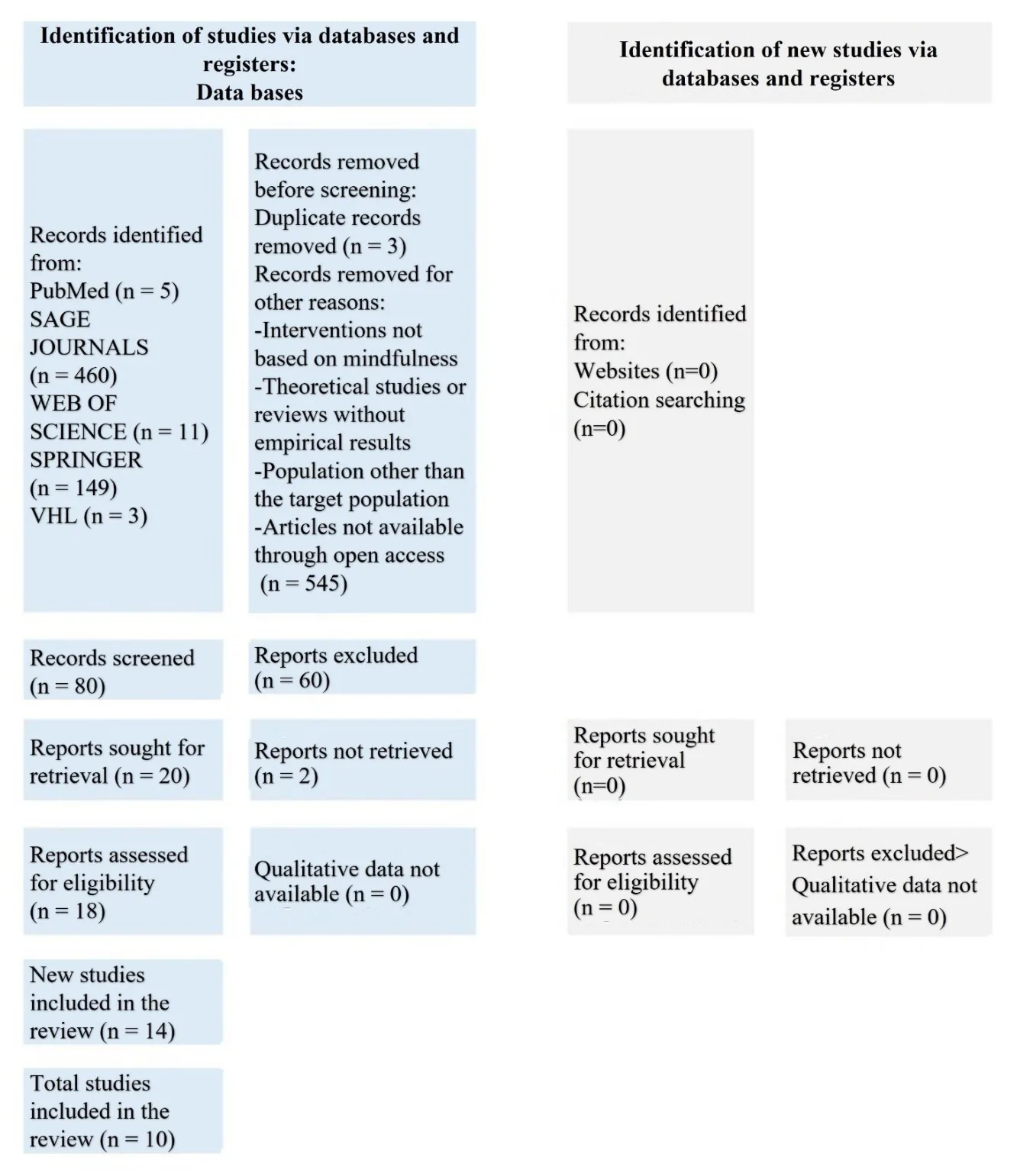
PRISMA ScR flow diagram for the identification of studies via databases and registers

With respect to educational level, few studies reported this information. Among those that did, most participants had completed secondary education. Finally, the duration of incarceration was reported inconsistently across studies, ranging from relatively short periods of 8 months to as long as 23 years Table 1.

**Table 1:** Characteristics of the study population (2015-2025)

Author/year	Country	Sex	Age	Marital status	Educational level	Mean duration of incarceration	Study design
Ferstz et al.^29^, 2015	United States	Women	20-53 years	Single	Not applicable	Not applicable	QE
Himelstein et al.^30^, 2015	United States	Men	14-18 years	Not applicable	Not applicable	8 months	RCT
Xu et al.^31^, 2016	China	Men	22-57 years	Married	Secondary	Not applicable	QE
An et al.^32^, 2019	China	Men	19-57 years	Not applicable	Not applicable	3 years	QE
Song et al.^33^, 2021	China	Women	40 years	Not applicable	Secondary	15 years	QE
Bouw et al.^34^, 2019	Netherlands	Men	23-55 years	Not applicable	Not applicable	15 -209 months	QE
Simpson et al.^35^, 2019	United Kingdom	Men	16-21 years	Not applicable	Secondary	52 months	QE
Davies et al.^36^, 2021	United Kingdom	Men and women	26-35 years	Not applicable	Not applicable	23 years	QE
Gallego et al.^37^, 2023	Spain	Men	23-58 years	Not applicable	Not applicable	Not applicable	RCT
Carmo et al.^38^, 2024	Portugal	Men	23-60 years	Single	Secondary	Not applicable	QE

QE=Quasi-experimental study, RCT = Randomized controlled trial

The selected studies were analyzed systematically, and their findings were classified into two main domains: (1) study design and intervention components, and (2) study results and the effects of the intervention on stress, anxiety, and depression.

**Characteristics of the identified intervention designs**

**Quasi-experimental studies:** Eight quasi-experimental studies^29^,^31^-^36^,^38^ and two randomized controlled trials^30^,^37^ were identified that evaluated mindfulness interventions for reducing stress, anxiety, and depression among incarcerated individuals.

Among the quasi-experimental studies, interventions were delivered in person and included between 18 and 44 participants per study group. Intervention duration ranged from 6 to 12 weeks. Most interventions comprised 6 to 12 sessions, with each session lasting approximately 1 to 3 hours. Activities implemented in the intervention groups included meditation, relaxation exercises, body scan, seated meditation, yoga, mindful movement, and group discussions^29^-^38^. Regarding the care provided to the control groups, several studies did not describe the comparator intervention^29^,^31^-^33^,^35^,^38^. Others used alternative interventions, such as substance use treatment programs^30^, individual psychotherapy^33^, or sports programs or training provided after the study period^32^,^37^. The findings showed significant reductions in stress^29^,^33^, anxiety, and depression following the intervention^29^,^31^,^34^-^36^,^38^, as well as improvements in self-esteem^30^,^32^ and sleep quality^29^.

**Randomized controlled trials**

In contrast, the randomized controlled trials included between 33 and 44 participants per study group. The interventions lasted 8 and 10 months, respectively, and consisted of 8 to 11 sessions of approximately 2 hours each. The interventions comprised meditation practices, psychoeducation, practical exercises, guided meditation, and informal practice, together with the use of workbooks. Regarding the control groups, mindfulness-based interventions were provided after the trial period^30^,^37^. The findings indicated that mindfulness-based interventions produced significant reductions in stress, anxiety, and depression, with effect sizes ranging from moderate to large^30^,^37^ Table 2.

**Table 2:** Overall characteristics of the intervention designs identified between 2015 and 2025

Author/year	Sample	Mode of delivery	Duration	Number of sessions and length	Intervention group care	Control group care	Primary outcomes	Findings
Ferstz, et al.^29^, 2015	Intervention group (IG): 18 participants; Control group (CG): 15 participants	Face-to-face	12 weeks	12 sessions; 1 hour per session	Meditation, relaxation exercises, and mindful breathing	Treatment not reported	Perceived stress decreased from 24.61 to 20.83 (t (17) = 2.805, p = 0.012); anxiety, measured with the State-Trait Anxiety Inventory (STAI), decreased from 55.44 to 43.11 (t (17)= 4.079, p = 0.001); and depression, assessed using the Center for Epidemiologic Studies Depression Scale-10 (CES-D-10), decreased from 17.78 to 13.56 (t (17) = 4.228, p = 0.001).	The mindfulness intervention significantly improved sleep quality, as reflected by a reduction in the mean Pittsburgh Sleep Quality Index (PSQI) score from 12.80 to 9.80 (t (14) = 3.574, p = 0.003).
Himelstein et al.^30^, 2015	IG: 27 participants; CG: 27 participants	Face-to-face	12 weeks	12 sessions; 15-25 minutes per session	Mindfulness program (mindfulness meditation and individual counseling)	Substance use treatment program and individual psychotherapy	The mindfulness intervention significantly improved decision-making skills from 8.8 (SD= 2.0) to 10.1 (SD = 1.8), t = −3.44, p < 0.01. Attitudes toward drugs (pre-intervention M= 38.7, SD = 5.4; post-intervention M = 40.7, SD = 7.2; t = −1.60) and mindfulness (Mindful Attention Awareness Scale [MAAS]: pre-intervention M = 58.8, SD = 10.9; post-intervention M = 60.5, SD = 11.1; t = −0.90) changed in the expected direction toward greater well-being.	Participants who received mindfulness training experienced a significant increase in self-esteem, from 21.6 (SD = 3.7) to 22.8 (SD =3.8), t = −2.07, p < 0.05.
Xu et al.^31^, 2016	IG: 19 participants; CG: 21 participants	Face-to-face	6 weeks	6 sessions; 2.5-3 hours per session	Body scan, seated meditation, walking meditation, yoga, and group discussions	Treatment not reported	Significant group-by-time interactions were observed for anxiety, depression, tension-anxiety, depression-dejection, anger-hostility, confusion-bewilderment, and total mood disturbance, with significant improvements from pre- to post-intervention in the mindfulness training group, whereas the control group showed no meaningful changes. For example, significant reductions were observed for tension-anxiety (F (1.38) = 24.79, p < 0.001) and anger-hostility (F (1.38) = 22.02, p < 0.001).	Simple-effects analyses showed that the mindfulness group had significantly higher mindfulness scores at posttest than the control group (F (1.38) = 12.91, p < 0.001), whereas no significant changes were observed in the control group (F (1.38) = 0.25, p = 0.62).
An et al.^32^, 2019	IG: 39 participants; CG: 44 participants	Face-to-face	6 weeks	6 sessions; 2.5-3 hours per session	PMeditation practices and mindfulness yoga	Received the same mindfulness training after the posttrial period	Compared with the waitlist control group, participants who received mindfulness training showed significant improvements in several psychological indicators. These included increased mindfulness (p < 0.01), significant reductions in Symptom Checklist-90 (SCL-90) scores (p < 0.001) and aggression (p < 0.05), as well as improvements in sleep quality (p < 0.05) and reductions in total mood disturbance (p < 0.01).	In addition, when participants were compared according to sentence length, mindfulness training was more effective among individuals serving longer prison sentences, who showed significant improvements in mindfulness (p < 0.05) and SCL-90 scores (p < 0.001), along with reductions in anxiety, depression, aggression, perceived stress, and total mood disturbance (all p < 0.05).
Song et al.^33^, 2021	IG: 25 participants; CG: 30 participants	Face-to-face	8 weeks	8 sessions; 2 hours of theory and 30-40 minutes of practice per session	Body scan, seated meditation and other audio-guided meditations, together with informal practice (purposefully paying attention to routine daily activities)	Mindfulness-based cognitive therapy theory without practice	The results showed that standard Mindfulness-Based Cognitive Therapy (MBCT) significantly reduced perceived stress (p = 0.01, d = 0.65, moderate effect), depression (p = 0.002, d = 0.82, large effect), and state anxiety (p = 0.02, d = 0.59, moderate effect). However, the reduction in trait anxiety was not significant (p = 0.02, d = 0.48, small effect).	Standard MBCT was more effective than theoretical instruction alone in reducing stress, state anxiety, and depression, although its impact on trait anxiety was limited.
Bouw et al.^34^, 2019	IG: 22 participants; CG: 10 participants	Face-to-face	8 weeks	8 sessions; 2 hours per session	Mindfulness exercises (body scan, object-focused meditation, breathing exercises, and slow-movement yoga)	Treatment not reported	During the mindfulness intervention, participants experienced positive changes in several emotional domains. Anger decreased significantly, with the median score declining from 3.0 before the intervention to 2.0 afterward (z = −2.151, p = 0.031), and mean anger intensity decreased from 6.6 (SD = 1.7) to 5.4 (SD = 1.9; t (15) = 2.30, p = 0.036, d = 0.69). Regarding stress, no significant changes were observed in its frequency (pre-intervention median = 4.0 vs. post-intervention median = 3.0; z = −1.458, p = 0.145), although mean stress intensity decreased from 7.5 (SD = 2.3) to 5.4 (SD = 2.7; t (18) = 4.16, p = 0.001, d = 0.82).	Participants' self-esteem increased significantly, from a mean of 7.4 (SD = 2.3) to 8.3 (SD = 1.2; t (21) = 2.82, p = 0.010, d = 0.53), indicating an enhanced positive self-perception.
Simpson et al.^35^, 2019	IG: 23 participants; CG: 23 participants	Face-to-face	10 weeks	10 sessions; 30–45 minutes per session	Breath awareness, body scan, and mindful movement	Treatment not reported	In terms of quantitative outcomes, the intervention produced positive effects on several measures. Impulsivity scores decreased significantly on both the Teen Conflict Survey TCS (d = 0.72; 95% CI: 0.32–1.11; p = 0.001) and the Barret Impulsivity Scale BIS-11 (d = 0.50; 95% CI: 0.21–0.76; p = 0.01). The BIS-11 subscales showed reductions in attentional impulsiveness (d = 0.44; p = 0.005), motor impulsiveness (d = 0.39; p = 0.04), and non-planning impulsiveness (d = 0.36; p = 0.01). Improvements were also observed in attention (d = 0.33; p = 0.01), cognitive instability (d = 0.46; p = 0.01), and self-control (d = 0.37; p = 0.02).	Mental distress decreased, with a small effect size (d = 0.32; 95% CI: 0.03–0.60; p = 0.26).
Davies et al.^36^, 2021	IG: 17 participants; CG: 16 participants	Face-to-face	10 months	8 sessions; 2 hours per session	Psychoeducation, group sharing of experiences, and practical exercises; sessions were supplemented with a workbook and between-session practice	Treatment not reported	Regarding perceived stress, participants showed a significant reduction in Perceived Stress Scale (PSS) scores (t (30) = 4.12, p = 0.001, d = 0.80), indicating a substantial decrease in psychological distress. Depression and anxiety symptoms also decreased significantly, as measured by the Self-Rating Depression Scale (SDS) (t (27) = 3.48, p = 0.002, d = 0.67) and the Self-Rating Anxiety Scale (SAS) (t (27) = 3.21, p = 0.003, d = 0.61), demonstrating a moderate effect of the intervention on psychological well-being.	Improvements were also observed in impulse control and sustained attention, as evidenced by a significant reduction in Stop-Signal Reaction Time (SSRT) (t (29) = −2.64, p = 0.013, d = 0.68), indicating greater cognitive control and lower impulsivity. Mindfulness levels also increased significantly following the intervention.
Gallego et al.^37^, 2023	IG: 22 participants; CG: 22 participants	Face-to-face	8 weeks	11 sessions; 2 hours per session	Formal meditation was practiced for 10 minutes during the first session, 15 minutes during the second session, and 20 minutes during subsequent sessions	Sports program	Regarding depression, the Wilcoxon signed-rank test showed a significant reduction in the mindfulness group between baseline and the 2-month follow-up (Z = −2.383; p = 0.017; r = −0.53). Anxiety scores decreased from moderate to mild levels at the 2-month follow-up, although the overall change did not reach statistical significance (χ² = 10.73; p = 0.067).	The most pronounced changes were observed for stress. In the mindfulness group, stress levels decreased significantly from moderate to normal (χ² = 16.44; p = 0.032), with significant differences between baseline and post-intervention (Z = −2.609; p = 0.009; r = −0.56) and between baseline and the 2-month follow-up (Z = −2.533; p = 0.011; r = −0.57), indicating a large and sustained effect.
Carmo et al.^38^, 2024	IG: 22 participants; CG: 22 participants	Face-to-face	5 months	18 sessions; 90 minutes per session	Mindfulness meditation exercises (mindful yoga, body scan, seated meditation, and mindful walking)	Treatment not reported	Following the intervention, although no statistically significant between-group differences were observed in stress, anxiety, or depression, mean scores were consistently lower in the mindfulness group. Self-esteem remained significantly higher in the mindfulness group (M = 32.42, SD = 5.96) than in the control group (M = 28.55, SD = 4.47; U = 140, p = 0.016, d = 0.35), with a small but consistent effect.	At baseline, the control group had higher levels of anxiety (U = 152.5, p = 0.034, d = 0.25) and negative affect (U = 130.5, p = .009, d = 0.35), whereas the mindfulness group demonstrated higher self-esteem (U = 158, p = 0.048, d = 0.31).

Face-to-face = in person. IG: Intervention Group; CG: Control Group; SD: Standard Deviation, M: Mean; CI: Confidence

**Effective mindfulness interventions for reducing stress, anxiety, and depression**

The overall analysis of mindfulness interventions identified a total of eight studies that evaluated the effectiveness of different programs for reducing stress, anxiety, and depression among incarcerated individuals^29^,^31^-^38^. The interventions included Path of Freedom^29^,^36^. Mindfulness-Based Cognitive Therapy (MBCT), and Mindfulness-Based Stress Reduction (MBSR) ^31^-^34^,^36^-^38^.

Regarding the number of sessions, most interventions were delivered in a group format and ranged from 2 to 18 sessions. Session duration ranged from 1 to 3 hours. All interventions were delivered by certified mindfulness instructors with experience in mindfulness, underscoring the importance of qualified personnel to ensure intervention effectiveness. 

The most important results showed that all eight interventions achieved significant reductions in stress, anxiety, or depression^29^,^31^,^32^,^34^-^38^. The most effective interventions were the adapted Path of Freedom program^29^,^36^ and MBCT^31^,^32^,^34^,^36^. These interventions produced significant reductions in anxiety, depression, and perceived stress (p < 0.01), with moderate-to-large effect sizes (d = 0.82 to 0.84)^32^,^36^.

The 12-session Path of Freedom intervention^29^ achieved significant reductions in stress (p = 0.012), anxiety (p = 0.001), and depression (p = 0.001). The 6-session MBCT intervention^31^ produced significant reductions in anxiety (p = 0.001) and depression (p = 0.024). MBCT protocols^34^,^36^ also demonstrated reductions in anxiety, depression, and total mood disturbance (p < 0.001). The 18-session intervention combining MBSR and MBCT^38^ showed a positive effect in reducing anxiety (p = 0.034), although the effect size was small (d = 0.25) Table 3.

**Table 3:** Effective mindfulness interventions for reducing stress, anxiety, and depression

Author/year	Intervention name	Delivery/ components	Setting/sessions	Intervention vs. Control Outcome
Ferstz, et al.^29^, 2015	Adapted Path of Freedom intervention	Certified mindfulness instructor accredited by the Prison Mindfulness Institute	Correctional facility; 12 group sessions; 1 hour per session	A significant reduction in perceived stress was observed following the intervention (post-intervention M = 20.83, d = 1.48) compared with baseline (pre-intervention M = 24.61, d = 0.95), t (17) = 2.805, p = 0.012. Anxiety also decreased significantly, with post-intervention scores (M = 43.11, d = 1.46) being lower than baseline scores (M = 55.44, d = 2.45), t (17) = 4.079, p = 0.001. Likewise, participants showed a significant improvement in depression, with mean scores decreasing from M = 17.78 (d = 1.20) pre-intervention to M = 13.56 (d = 1.17) post-intervention, t (17) = 4.228, p = 0.001.
Xu et al.^31^, 2016	Mindfulness-Based Cognitive Therapy (MBCT) protocol	Mindfulness instructor with experience	Correctional facility; 6 group sessions; 2.5–3 hours per session	Significant time-by-treatment interactions were observed for anxiety and depression (p < 0.05). Simple-effects analyses showed that the mindfulness group experienced significant reductions in anxiety (SAS: F (1.38) = 13.75, p = 0.001) and depression (SDS: F (1.38) = 5.63, p = 0.024) at posttest compared with pretest. Significant reductions were also observed in tension-anxiety (F (1.38) = 19.42, p < 0.001), depression-dejection (F (1.38) = 7.42, p = 0.01), anger–hostility (F (1.38) = 13.59, p < 0.001), confusion-bewilderment (F (1.38) = 8.92, p = 0.005), and total mood disturbance (TMD: F (1.38) = 12.42, p < 0.001).
An et al.^32^, 2019	Mindfulness-Based Stress Reduction (MBSR) and Mindfulness-Based Cognitive Therapy (MBCT)	Mindfulness instructor with experience	Correctional facility; 6 group sessions; 2.5–3 hours per session	The mindfulness group showed significant reductions in overall psychological distress (p = 0.000), particularly in symptoms of somatization (p = 0.002), obsessive–compulsive symptoms (p = 0.000), interpersonal sensitivity (p = 0.000), depression (p = .000), anxiety (p = 0.001), hostility (p = 0.000), phobic anxiety (p = 0.006), paranoid ideation (p = 0.001), and psychoticism (p = 0.002).
Song et al.^33^, 2021	Mindfulness-Based Cognitive Therapy (MBCT) protocol	Mindfulness instructors certified by the Oxford Mindfulness Center	Correctional facility; 8 group sessions; 2 hours plus 30–40 minutes of practice per session	An analysis of covariance (ANCOVA) was performed to control for pretest values and evaluate the effects of the intervention. Significant between-group differences were found for state anxiety (F (2.63) = 3.28, p = 0.04) and depression (F (2.63) = 6.37, p < 0.001). Post hoc analyses showed that the standard MBCT group had significantly lower levels of state anxiety than the theoretical learning group (t = 2.55, p = 0.04, d = 0.67). Likewise, depression levels in the standard MBCT group were significantly lower than those in the waitlist control group (t = 3.57, p = 0.002, d = 0.84).
Bouw et al.^34^, 2019	Mindfulness-Based Cognitive Therapy (MBCT) protocol	Mindfulness instructor with experience	Correctional facility; 2 group sessions; 2 hours per session	The reduction in stress intensity (from 7.5 to 5.4) was statistically significant, with a moderate-to-large effect size (d = 0.82).
Davies et al.^36^, 2021	Adapted 12-session Path of Freedom intervention	Mindfulness instructor	Correctional facility; 2 group sessions; 2 hours per session	A significant increase in mindfulness was observed following the intervention, as reflected by Five Facet Mindfulness Questionnaire (FFMQ) scores (F = 8.86, p = 0.006) and Child and Adolescent Mindfulness Measure–Short Form (CAMSR) scores (F = 5.69, p = 0.024). Perceived stress also decreased significantly over time (F = 19.177, p < 0.001; η²P = 0.398).
Gallego et al.^37^, 2023	Mindfulness-Based Cognitive Therapy (MBCT) protocol	Mindfulness instructor with experience	Correctional facility; 11 group sessions; 2 hours per session	For stress, significant differences were observed across assessment time points (χ²(3) = 8.793, p = 0.032), with a marked reduction following the intervention (p = 0.009, r = −0.556), which was maintained at the 2-month follow-up (p = 0.011, r = −0.567). For anxiety, no overall significant differences were found (χ²(3) = 4.310, p = 0.230), although a moderate reduction was observed at the 2-month follow-up (p = 0.036, r = −0.468). Likewise, no overall significant changes were found for depression (χ²(3) = 6.241, p = 0.100); however, depression scores were significantly lower at the 2-month follow-up (p = 0.017, r = −0.533). Although a downward trend was observed immediately after the intervention, it did not reach statistical significance (p = 0.080, r = −0.392).
Carmo et al.^38^, 2024	Mindfulness-Based Stress Reduction (MBSR) and Mindfulness-Based Cognitive Therapy (MBCT)	Mindfulness instructor certified in MBCT	Correctional facility; 18 group sessions; 90 minutes per session (60 minutes of meditation practice and 30 minutes of feedback)	The mindfulness intervention showed positive effects in reducing anxiety, with participants in the mindfulness group demonstrating significantly lower anxiety levels than those in the control group (M = 2.82, SD = 3.81 vs. M = 4.95, SD = 4.69; U = 152.50, p = 0.034). However, the effect size was small (d = 0.25).

M: Mean; SD: Standard Deviation; SAS: Self-Rating Anxiety Scale; SDS: Self-Rating Depression Scale

## Discussion

The studies included in this review provide evidence supporting the effectiveness of mindfulness in correctional settings, although they predominantly involved samples of incarcerated men. This limits the understanding of its impact among incarcerated women, who may respond differently to the intervention^38^. These differences may be attributable to variations in emotional regulation and in the prevalence of psychological disorders such as depression and anxiety. Additional studies involving female or mixed-sex samples are needed to evaluate potential sex differences in the effectiveness of mindfulness interventions^39^.

Another relevant aspect is the variability in participants' age. Some studies suggest that younger adults may show greater initial resistance to mindfulness practice, whereas older adults tend to engage more readily and derive greater benefits because of a greater willingness to engage in emotional learning^40^. These findings suggest that the effects of mindfulness interventions may vary according to age, highlighting the need to tailor intervention strategies to the age-related characteristics of the incarcerated population.

In addition, the length of incarceration appears to play a key role in incarcerated individuals' willingness to participate in mindfulness programs. Studies have suggested that individuals in the early years of their sentence tend to experience higher levels of stress and anxiety, which may make them more receptive to this type of intervention. In contrast, those who have spent longer periods in prison may be more resistant to change or less motivated to engage in emotional regulation programs^41^.

A central issue emerging from this review is the differential effectiveness of the interventions evaluated. Among them, the adapted Path of Freedom program demonstrated the greatest effectiveness in reducing stress, anxiety, and depression. This result suggests that its impact extends beyond traditional mindfulness practices by more systematically integrating key components of emotional regulation^40^. Unlike other protocols that focus exclusively on meditation and mindfulness practice, Path of Freedom incorporates emotional intelligence strategies, interpersonal skills development, and behavioral management, which may enhance its therapeutic effects in correctional populations^42^.

From a theoretical perspective, combining mindfulness with elements of emotional intelligence may explain the greater effectiveness of this intervention. Mindfulness alone reduces stress and anxiety by fostering greater present-moment awareness and improving the regulation of automatic responses to adverse situations^43^. However, the inclusion of skills such as emotion identification and regulation, enhanced decision-making, and strengthened self-control may represent a key differentiating factor in reducing depression. In correctional settings, where individuals are exposed to high levels of emotional distress and interpersonal conflict, the ability to regulate emotions effectively becomes essential for psychological well-being^44^.

Likewise, the structured design of Path of Freedom, which includes group sessions led by certified instructors and an interactive methodology combining meditation practice, psychoeducation, and self-reflection activities, may promote greater adherence to the intervention^45^. This holistic approach may explain why its effects were more consistent across all psychological variables evaluated than those of shorter programs or interventions with less methodological diversity^46^.

Among the strategies evaluated, MBSR and MBCT were used in five studies and demonstrated variable effectiveness in reducing stress, anxiety, and depression, with effect sizes ranging from low to moderate^47^. MBSR focuses on stress reduction and the promotion of overall well-being through practices such as meditation, body scan, and yoga, making it a useful approach for enhancing emotional regulation across diverse populations, including incarcerated individuals^48^.

In contrast, MBCT combines mindfulness with principles of CBT, focusing on modifying negative thought patterns to prevent depressive relapse, making it more suitable for individuals with a history of depression^48^. Whereas MBSR aims to strengthen resilience to stress^49^, MBCT emphasizes cognitive restructuring. In correctional settings, the choice between these interventions should be guided by participants' psychological profiles, and combining both interventions may optimize their benefits by providing a more comprehensive approach^49^.

## Conclusions

Mindfulness-based interventions have been shown to be effective in reducing stress, anxiety, and depression among incarcerated individuals. However, their effectiveness varies according to the intervention approach, program duration, and the components incorporated. The Path of Freedom intervention demonstrated the greatest effectiveness, possibly because of its structured integration of mindfulness with emotional intelligence and interpersonal skills.

Factors such as sex, age, educational level, and length of incarceration may influence adherence to and the effectiveness of these interventions, underscoring the need for more targeted research. Among the interventions evaluated, both MBSR and MBCT have demonstrated benefits, although through different approaches. MBSR focuses on stress regulation and the promotion of overall well-being, whereas MBCT emphasizes cognitive restructuring to prevent depressive relapse. Combining these strategies may enhance their therapeutic effects by providing a more comprehensive approach in correctional settings.
